# Extracting Physicochemical Features to Predict Protein Secondary Structure

**DOI:** 10.1155/2013/347106

**Published:** 2013-05-14

**Authors:** Yin-Fu Huang, Shu-Ying Chen

**Affiliations:** Department of Computer Science and Information Engineering, National Yunlin University of Science and Technology, 123 University Road, Section 3, Touliu, Yunlin 640, Taiwan

## Abstract

We propose a protein secondary structure prediction method based on position-specific scoring matrix (PSSM) profiles and four physicochemical features including conformation parameters, net charges, hydrophobic, and side chain mass. First, the SVM with the optimal window size and the optimal parameters of the kernel function is found. Then, we train the SVM using the PSSM profiles generated from PSI-BLAST and the physicochemical features extracted from the CB513 data set. Finally, we use the filter to refine the predicted results from the trained SVM. For all the performance measures of our method, *Q*_3_ reaches 79.52, SOV94 reaches 86.10, and SOV99 reaches 74.60; all the measures are higher than those of the SVMpsi method and the SVMfreq method. This validates that considering these physicochemical features in predicting protein secondary structure would exhibit better performances.

## 1. Introduction

Many issues on molecular biology have been addressed in the past decades, including genetics, structural biology, and drug design. A protein primary sequence is composed of amino acids; as we know, totally 20 different kinds of amino acids can be found in protein sequences. In this paper, we would investigate protein secondary structures based on protein sequences.

The secondary structure of a protein sequence comes from different folding of amino acids, due to the differences of their side chain sizes, shapes, reactivity, and the ability to form hydrogen bonds. Furthermore, owing to the differences of the side chain sizes, the number of electric charges, coupled with the affinity for water, the tertiary structures of protein sequences are not all the same. Thus, the exploration of molecular structures on protein sequences is divided into secondary, tertiary, and even quaternary structures. Given a protein primary sequence, its corresponding secondary structure can be revealed as follows:

 Primary sequence:
 MFKVYGYDSNIHKCVYCDNAKRLLTVKKQPFEFINIMPEKGV
 Secondary structure:
 CEEEEECCCCCCCCHHHHHHHHHHHHCCCCEEEEECCCCTTC.

A protein sequence affects the structure and function; in other words, a protein sequence determines its structure, and the structure determines functions. If amino acids in a protein sequence are arranged in a different order in the skeleton branch of the side chain R group, the nature of the protein would reveal specific functions. Even for different species of proteins, if they have a similar structure, their functions would be also similar. Therefore, predicting the protein structure is crucial to the function analysis. Besides, the secondary structure refers to the relative position of the space between the atoms of a certain backbone. Traditional protein structure determination was done by protein X-ray crystallography or nuclear magnetic resonance (NMR). However, all experimental analysis costs much time. In order to shorten the time to help biologists, protein structure prediction by computers facilitates reaching this goal.

The prediction of protein secondary structure has been studied for decades. Early, the statistical analysis of secondary structure was done for a single amino acid. The most representative is the Chou and Fasman method [1], and the accuracy is only 50%. Next, the statistical analysis for amino acid segments was done further. A segment length is usually with 9~21 amino acids. Based on an amino acid segment, predicting the structure of central residues enables promoting the accuracy. The most representative is the GOR method [2], and the accuracy increases more than 10% (about 63%). At present, the prediction methods on protein secondary structure have evolved into using the PSI-BLAST program [3] to find the protein homology information, based on PSSM (position-specific scoring matrices) profiles. The accuracy of using PSSM to predict secondary structure has reached between 70 and 80% [4–7]. However, we believe that there still exists a great improvement in predicting protein secondary structure.

The rest of this paper is organized as follows. In Section 2, basic concepts used in the proposed methods are introduced first. In Section 3, we propose the methods and relevant features to predict the secondary structure of a protein sequence. Then, we make use of window sizes and tune parameters in the experiments in Section 4, in order to obtain better experimental results. Finally, we make a conclusion in Section 5.

## 2. Basic Concepts

### 2.1. Protein Secondary Structure

Protein secondary structure derived from the experimentally determined 3D structure has been defined using DSSP (Dictionary of Secondary Structures of Proteins) [8], STRIDE (STRuctural IDEntification) [9], and DEFINE (DEFINE_structure) [10]. DSSP is selected here so that our method can be compared with most existing methods, based on the same protein secondary structure definition. Eight secondary structure classes were defined there, that is, H(*α*-helix), G(310-helix), I(*π*-helix), E(*β*-strand), B(isolated *β*-bridge), T(turn), S(bend), and -(rest). The eight structure classes are usually reduced to three classes of helix (H), sheet (E), and coil (C). Five reductions could be performed as follows:

1. H, G and I to H; E to E; the rest to C
2. H, G to H; E, B to E; the rest to C
3. H, G to H; E to E; the rest to C
4. H to H; E, B to E; the rest to C
5. H to H; E to E; the rest to C.

The first reduction was used in the PHD (Profile network from HeiDelberg) method [7] which is the early secondary structure prediction method using multiple sequence alignments of proteins homologous with a query protein sequence. We also use the first reduction in order to provide a fair comparison with other prediction methods.

### 2.2. SVM (Support Vector Machine)

SVM was first investigated by Boser et al. in 1992 [11]. It solves linearly inseparable problems by nonlinearly mapping the vector in a low dimensional space to a higher dimensional feature space and constructs an optimal hyper-plane in the higher dimensional space. Therefore, SVM has high performances in data classification. A classification task usually involves with training and testing data which consist of some data instances. Each instance in the training set contains one “target value” (i.e., class label) and several “attributes” (i.e., features). The goal of SVM is to produce a model which can predict the target value of data instances in the testing set by using the attributes.

## 3. Methods and Features

### 3.1. System Architecture

**Figure 1:**
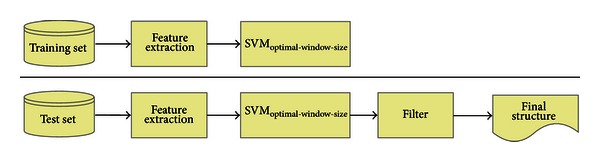
System architecture.

The system architecture of predicting protein secondary structure is divided into three steps, as illustrated in Figure 1. The first step is to determine/extract the relevant features in/from protein sequences. Then, in the second step, we feed the features into SVM, respectively, in the training and test phases. Finally, we use a filter method to refine the predicted results from the trained SVM. During the SVM training phase, we not only train the SVM using the training data, but also, in advance, find the optimal sliding window size and the cost and gamma parameters of SVM kernel function, using the entire data set. The details about each module in the system architecture are depicted in the following subsections.

### 3.2. Feature Extraction

**Figure 2:**
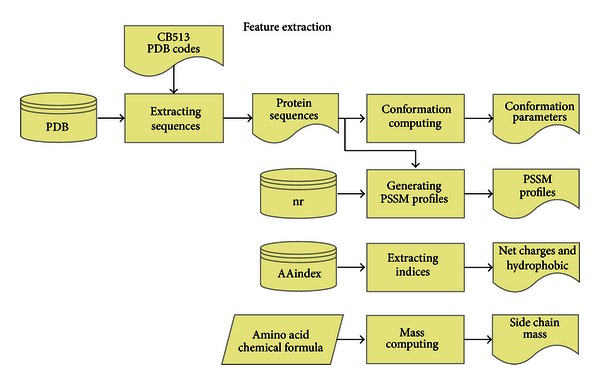
Process of feature extraction.

Five relevant kinds of features are extracted from protein sequences to predict protein secondary structure, that is, (1) conformation parameters, (2) position specific scoring matrix (PSSM) profiles, (3) net charge, (4) hydrophobic, and (5) side chain mass. The process of feature extraction is shown in Figure 2.

#### 3.2.1. Extracting Sequences

First, we extract amino acid and secondary structure sequences from the PDB website (http://www.rcsb.org/pdb/home/home.do), using the PDB codes of CB513 [12]. Then, we can further extract five different features from amino acid sequences as follows.

#### 3.2.2. Conformation Parameters

Conformation parameters are the proportions that residues (or amino acids) tend to secondary structure. In general, protein secondary structure is divided into three types: *α*-helix (H), *β*-sheet (E), and coil (C), so that there are three values for each amino acid. In the feature extraction, all the conformation parameters are calculated from a data set. The conformation parameters for each amino acid *S*_*ij*_ are defined as follows:

(1) $$\begin{array}{c} S_{ij}=\frac{a_{ij}}{a_{i}},\;\text{where}\;\;i=1,\ldots ,20,\;j=1,2,3. \end{array}$$

In this formula, *i* indicates the 20 amino acids, and*j* indicates the 3 types of secondary structure: H, E, and C. Here, *a*_*i*_ is the amount of the *i*th amino acid in a data set whereas *a*_*ij*_ is the amount of the *i*th amino acids with the *j*th secondary structure. The conformation parameters for each amino acid in a data set are shown in Table 1. The reason of using conformation parameters as features is that the folding of each residue has some correlation with forming a specific structure.

#### 3.2.3. PSSM Profiles

PSSM profiles are generated by PSI-BLAST (Position Specific Iterative-Basic Local Alignment Search Tool) program. Since PSSM profiles are involved with biological evolution, we consider them as features in our work. A PSSM profile has *L* × 20 elements, where *L* is the length of a query sequence. These profiles are then used as the input features to feed an SVM, employing a sliding window method.

PSI-BLAST is based on BLAST which has been published by Altschul et al. in 1997 [3]. Since PSI-BLAST program is more sensitive than other methods, we can find a lot of low similarity sequences and similarity structure function of protein sequences. First, a database containing all known sequences (or nonredundant database) is selected. Then, low complexity regions are removed from the nr database. Finally, PSI-BLAST program is used to query each sequence in CB513 and generates PSSM profiles after three iterations. Here, multiple sequence alignment (MSA) and BLOSUM62 matrix [13] are used in this process.

The reason of using the sliding window method is to get more surrounding information of residues. We consider a sliding window of size 7~19 at which a predicted residue is centered to extract input features. The optimal window size yielding favorable predictive performances would be obtained experimentally. For the *i*th residue centered at the sliding window of size 7, we can get 7 × 20 features *F*_*i*+*n*,*j*_ where *n* is in the range [−3, 3] and *j* is the PSSM column from 1 to 20.

#### 3.2.4. Net Charges

There are five amino acids with charges, that is, R, D, E, H, and K. Since residues with similar electric charges repel each other and interrupt the hydrogen bond of main chain, they are adverse to *α*-helix formation. Besides, the continuous residues of *β*-sheet cannot be with similar charges. This information facilitates predicting the secondary structure. The net charge of amino acids can be taken from Amino Acid index database (or AAindex) [14–18], as shown in Table 2. A plus sign represents a positive charge and a minus sign represents a negative charge.

#### 3.2.5. Hydrophobic

For protein folding, polar residues prefer to stay outside of protein to prevent non-polar (hydrophobic) residues from exposing to polar solvent, like water. Therefore, hydrophobic residues appearing periodically can be used for predicting protein secondary structure. In general, the residues in *α*-helix structure are made up of one segment of hydrophobic and one segment of hydrophilic. However, *β*-sheet structure is usually influenced by the environment, so this phenomenon is not obvious. In other words, hydrophobic affects the stability of secondary structure. The hydrophobic values of amino acids can also be obtained from Amino Acid index database (or AAindex) [14–18], as shown in Table 3. The more positive values are, the more hydrophobic is.

#### 3.2.6. Side Chain Mass

**Figure 3:**
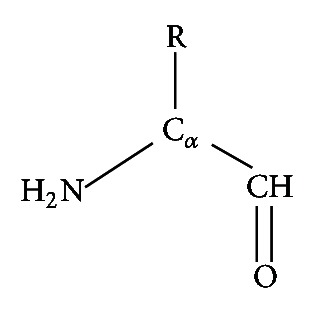
Basic structure of amino acids.

Although the basic structure as shown in Figure 3 is the same for 20 amino acids, the size of the side chain R group still influences structure folding. Here, we explain the influences as follows. First, the side chain R group is distributed in the outside of the main chain of *α*-helix structure, but the continuous large R groups can make *α*-helix structure unstable, thereby disabling amino acids from forming *α*-helix structure. Next, the R group with ring structure like proline (P) is not easy to form *α*-helix structure. Proline is composed of 5 atoms in a ring, which is not easy to reverse and is also not easy to generate a hydrogen bond. Finally, we observe that the R group of *β*-sheet structure is smaller than those of other structures, in general. Therefore, we include the side chain mass as a feature, as shown in Table 4.

### 3.3. SVM (Finding the Optimal Window Size and Parameters)

The SVM used in the experiments is a classifier for predicting the secondary structure H, E, and C. Threefold cross-validation is employed on the CB513 data set to find (1) the optimal window size in the range [7, 19] and (2) the optimal parameters of the kernel function, such as cost C and gamma *γ*. Here, the kernel function used in the SVM is RBF (i.e., Radial Basis Function). To solve the multiclass problem confronted in the work, we employ the “one-against-one” approach. For 3 classes, we need 3 binary classifiers and set the labels of the secondary structure (H, E, C) to (−1, +1, +2). Then, we use the max-wins voting strategy to determine the class; in other words, each binary classifier casts a vote, and the winning class is with the highest number of votes. In the experiments, the LIBSVM tool kit proposed by Chang and Lin [19] would be used to implement the program. After the optimal window size and parameters are found, we would use the SVM for training and test.

### 3.4. Filter

A single residue in its natural state cannot be alone folded into *α*-helix or *β*-sheet. Thus, setting thresholds on the length of consensus secondary structure can be used to filter out incorrect predicted results. For example, at least three contiguous residues are for *α*-helix and at least two contiguous residues are for *β*-sheet. For the current scanning window (*i* − 1, *i*, *i* + 1) in the predicted secondary structure, two possible structures could happen at position *i*:

Case H: if str(*i* − 1) and str(*i* + 1) are H, then str(*i*) is not changed; otherwise, extend the examined segment to (*i* − 3, *i* − 2, *i* − 1, *i*, *i* + 1, *i* + 2, *i* + 3) and replace str(*i*) with the majority structure in the examined segment.
Case E: if str(*i* − 1) or str(*i* + 1) is E, then str(*i*) is not changed; otherwise, extend the examined segment to (*i* − 3, *i* − 2, *i* − 1, *i*, *i* + 1, *i* + 2, *i* + 3) and replace str(*i*) with the majority structure in the examined segment.

**Figure 4:**
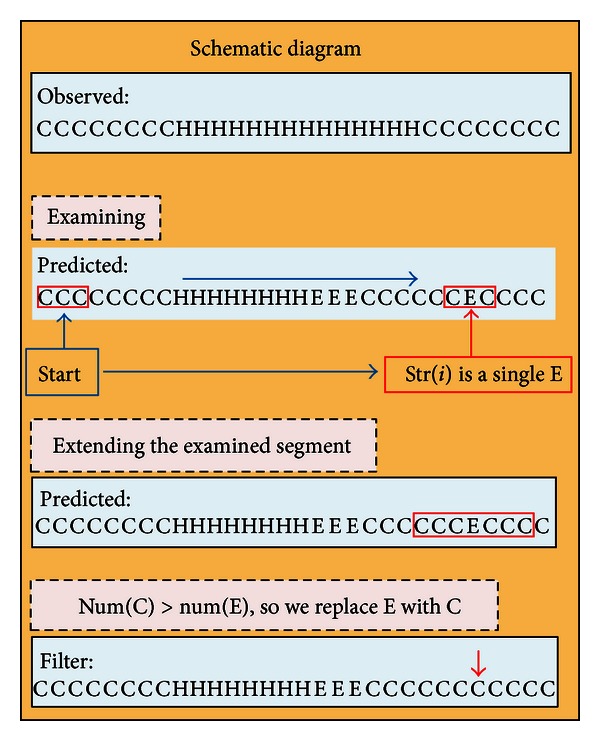
Schematic diagram for filtering 9INSb.

For the example as shown in Figure 4, after the filtering, *Q*_3_ for 9INSb is improved from 76.7 to 80 and SOV99 is improved from 77.8 to 93.3 where *Q*_3_ and SOV99 will be described in Section 4.2.

## 4. Experiments

### 4.1. Data Set

In the previous work, some typical data sets were frequently used in protein secondary structure prediction, such as RS126 [7], CB513 [12], CASP [20], and EVA [21]. Here, we consider the selected data set should be with low similarity; that is, the protein sequences within the data set are not similar to each other. Thus, the protein secondary structure prediction we develop would enable predicting an unknown protein sequence more accurately.

In our work, the data set we choose is nonhomologous CB513 data set constructed by Cuff and Barton and contains 513 protein chains. Almost all the sequences in the RS126 data set are also included in the CB513 data set. The CB513 data set contains 16 chains of ≦30 residues. Although very short chains would slightly decrease the accuracy for the hard definition of secondary structures, we still include them in the set for comprehensive study. We retrieve the CB513 data set from the website: http://paraschopra.com/projects/evoca_prot/index.php, which contains 84,093 residues where 34.59% of the residues is for helix, 21.35% for sheet, and 44.06% for coil, as shown in Table 5.

### 4.2. Performance Measures

Two kinds of performance measures are frequently used in protein secondary structure prediction; that is, *Q*_3_ or accuracy (three-state overall per-residue accuracy) and SOV99 [22] (or SOV94 [23]) (Segment Overlap measure). *Q*_3_ is a residue-based measure of three-structure overall percentage of correctly classified residues, which can be represented as

(2) $$\begin{array}{c} Q_{3}=\frac{{\text{H}}_{\text{pre}}+{\text{E}}_{\text{pre}}+{\text{C}}_{\text{pre}}}{N_{\text{total}}}, \end{array}$$

where *N*_total_ is the total number of predicted residues, H_pre_ is the correctly classified secondary structure for helix, E_pre_ for sheet, and C_pre_ for coil.

SOV99 is a segment-based measure of three structures, whose value is within the range [0, 100], as shown in Formula (3). SOV99 differs from *Q*_3_ in the prediction unit such that SOV99 would penalize wrong predictions; for example, a single helix predicted as a multiply-split helix is unrealistic prediction

(3) SOV=100×[1N∑i∈{H,E,C} ∑S(i)min⁡ov(s1,s2)+δ(s1,s2)max⁡ov(s1,s2)           × len(s1)],

where *s*_1_ and *s*_2_ denote segments of secondary structure *i* (H, E, or C), *S*(*i*) = {(*s*_1_, *s*_2_) : *s*_1_∩*s*_2_ ≠ *⌀*, *s*_1_ and *s*_2_ are both in structure  *i*}, *N* is a normalization value, min⁡*ov*(*s*_1_, *s*_2_) is the length of actual overlap of *s*_1_ and *s*_2_, max⁡*ov* (*s*_1_, *s*_2_) is the length of total extent for *s*_1_ and *s*_2_, and *δ*(*s*_1_, *s*_2_) can be represented as

(4) $$\begin{array}{c} \delta \left(s_{1},s_{2}\right)=\min\left\{\begin{array}{c} \max ov\left(s_{1},s_{2}\right)-\min ov\left(s_{1},s_{2}\right)\\ \min ov\left(s_{1},s_{2}\right)\\ \mathrm{int}\left(\frac{\text{len}\left(s_{1}\right)}{2}\right)\\ \mathrm{int}\left(\frac{\text{len}\left(s_{2}\right)}{2}\right). \end{array}\right\}. \end{array}$$

The definition of *δ* and the normalization value N are different for SOV99 and SOV94.

### 4.3. Optimal Parameters and Window Sizes

As introduced in Section 2.2, we adopt the well-known LIBSVM developed by Chang and Lin [19] as an SVM classifier. The kernel function used here is RBF (Radial Basis Function) since it is more accurate and effective than the other kernel ones. The parameters C and *γ* are determined by the optimum performance of 6 × 6 combinations between [2^0^,…, 2^5^] and [2^−6^,…, 2^−1^] for each window size. Moreover, the feature vector is normalized in the range [0, 1] and the number of features in a larger window size would become more. The optimal parameters and classification accuracy are evaluated in threefold cross-validation, as shown in Table 6.

According to the experimental results, we found the optimal parameters and window size are C = 2^1^, *γ* = 2^−4^, and WS = 13. Then, we use these parameters and window size to conduct the further experiments.

### 4.4. Experimental Results

In this section, we compare the experimental results without filtering and with filtering. For the classification results, a confusion matrix is employed to present the correct and false predictions based on the precision and recall, as shown in Tables 7 and 8. The precision and recall are expressed as follows:

(5) Precision(i)  =The number of correctly classified structure iThe number of total predicted structure i,                for   i=H,E,C,Recall(i)  =The  number  of  correctly  classified  structure  iThe  number  of  total  actual  structure  i,                for  i=H,E,C.

Obviously, the classification accuracy with filtering (i.e., 79.52%) is higher than that without filtering (i.e., 77.40%). The precision for H and the recall for C especially are improved from 83.64 to 92.00 (with filtering) and from 82.43 to 88.31 (with filtering), respectively. Therefore, the filter rules are required to improve the accuracy in predicting protein secondary structure.

### 4.5. Comparing with Other Methods

Here, we compare our methods with other four methods; that is, PHD, SVMfreq, PMSVM, and SVMpsi as shown in Table 9. Both the PHD and SVMfreq methods are based on the frequency profiles with multiple sequence alignment; however, the classifier used in the PHD method is a neural network (or NN) whereas the classifier used in the SVMfreq method is a support vector machine (or SVM). Similarly, both the PMSVM and SVMpsi methods are based on the PSSM profiles generated from PSI-BLAST. Although they use the same-type classifier (or SVM), the former adopts one-versus-one classifier (i.e., H/E, E/C, C/H) and the latter adopts the one-versus-rest classifier (i.e., H/~H, E/~E, C/~C).

As shown in Table 9, we found that all the performance measures of our method (i.e., the version with filtering), including *Q*_3_, SOV94, and SOV99, are higher than those of the other four methods, regardless using the CB513 or RS126 data sets. *Q*_3_ for the version with filtering (or without filtering) is improved by 2.92 (or 0.8), SOV94 for the version with filtering (or without filtering) is improved by 6 (or 10.1), and SOV99 for the version with filtering is improved by 1.1, compared with the results of the SVMpsi method for CB513 (i.e., the next best one).

However, our method (i.e., the version with filtering) has lower R(H) than the SVMpsi method (i.e., 76.91 versus 78.1). One of the possible reasons is that the threshold on the length of consensus secondary structure (i.e., at least three contiguous residues for H) is set in the filter. Although the recall for H is decreased, the predicted structures are more structurally meaningful. Besides, we found that two SOV measures in the SVMpsi and our methods vary greatly. Although SOV94 is decreased (i.e., from 90.20 to 86.10) after applying the filter in our method, the latest definition (i.e., SOV99) is still the highest.

## 5. Conclusions

In this paper, we propose a protein secondary structure prediction method using PSSM profiles and four physicochemical features, including conformation parameters, net charges, hydrophobic, and side chain mass. In the experiments, the SVM with the optimal window size and the optimal parameters of the kernel function is found first. Then, we train the SVM using the PSSM profiles and physicochemical features extracted from the CB513 data set. Finally, we use the filter to refine the predicted results from the trained SVM. For the experimental results, *Q*_3_, SOV94, SOV99, and recall of our method are higher than those of the SVMpsi method based on the PSI-BLAST profiles as well as the SVMfreq method based on the frequency profiles with multiple sequence alignment for the CB513 data set. In summary, considering these physicochemical features in predicting protein secondary structure would exhibit better performances.

## Figures and Tables

**Table 1:** Conformation parameters for each amino acid in a data set.

Amino acids	H	E	C
A	0.49	0.16	0.35
R	0.42	0.19	0.39
N	0.27	0.13	0.6
D	0.31	0.11	0.58
C	0.26	0.29	0.45
E	0.49	0.15	0.36
Q	0.46	0.16	0.38
G	0.16	0.14	0.7
H	0.3	0.22	0.48
I	0.35	0.37	0.28
L	0.45	0.24	0.31
K	0.4	0.17	0.43
M	0.44	0.23	0.33
F	0.35	0.3	0.35
P	0.18	0.09	0.74
S	0.28	0.19	0.54
T	0.25	0.27	0.48
W	0.37	0.29	0.35
Y	0.34	0.3	0.36
V	0.3	0.41	0.29

**Table 2:** Net charge of amino acids.

Amino acids	Mass
A	0
R	+1
N	0
D	−1
C	0
E	−1
Q	0
G	0
H	+1
I	0
L	0
K	+1
M	0
F	0
P	0
S	0
T	0
W	0
Y	0
V	0

**Table 3:** Hydrophobic values of amino acids.

Amino acids	Mass
A	1.8
R	−4.5
N	−3.5
D	−3.5
C	2.5
E	−3.5
Q	−3.5
G	−0.4
H	−3.2
I	4.5
L	3.8
K	−3.9
M	1.9
F	2.8
P	−1.6
S	−0.8
T	−0.7
W	−0.9
Y	−1.3
V	4.2

**Table 4:** Side chain mass of amino acids.

Amino acids	Mass
A	15.0347
R	100.1431
N	58.0597
D	59.0445
C	47.0947
E	73.0713
Q	72.0865
G	1.0079
H	81.0969
I	57.1151
L	57.1151
K	72.1297
M	75.1483
F	91.1323
P	41.0725
S	31.0341
T	45.0609
W	130.1689
Y	107.1317
V	43.0883

**Table 5:** Structures of the CB513 data set.

Structures	H	E	C	Total
Residues	29090	17950	37053	84093

**Table 6:** Optimal parameters for different window sizes.

Window sizes	Features	Best *C*	Best *γ*	Accuracy (%)
7	146	2^0^	2^−3^	76.3203
9	186	2^1^	2^−4^	76.7935
11	226	2^0^	2^−4^	77.4464
13	266	2^1^	2^−4^	78.0029
15	306	2^1^	2^−4^	77.7806
17	346	2^1^	2^−5^	77.6549
19	386	2^1^	2^−4^	77.5796

**Table 7:** Confusion matrix without filtering.

Actual	Predicted			
	H	E	C	Recall (%)
H	22976	931	5183	78.98
E	1044	11569	5337	64.45
C	3451	3059	30543	82.43
Precision (%)	83.64	74.36	74.38	77.40

**Table 8:** Confusion matrix with filtering.

Actual	Predicted			
	H	E	C	Recall (%)
H	22372	818	5900	76.91
E	432	11776	5742	65.60
C	1514	2819	32720	88.31
Precision (%)	92.00	76.40	73.76	79.52

**Table 9:** Comparisons between ours and other methods.

Methods	*Q*_3_	SOV94	SOV99	R(H)	R(E)	R(C)
PHD (RS126) [7]	70.8	73.5	—	72.0	66.0	72.0
SVMfreq (RS126) [5]	71.2	74.6	—	73.0	58.0	73.0
SVMfreq (CB513) [5]	73.5	76.2	—	75.0	60.0	79.0
PMSVM (CB513) [4]	75.2	80.0	—	80.4	71.5	72.8
SVMpsi (RS126) [6]	76.1	79.6	72.0	77.2	63.9	81.5
SVMpsi (CB513) [6]	76.6	80.1	73.5	78.1	65.6	81.1
Ours without filtering (CB513)	77.40	90.20	71.10	78.98	64.45	82.43
Ours with filtering (CB513)	79.52	86.10	74.60	76.91	65.60	88.31
